# Highly N-doped microporous carbon nanospheres with high energy storage and conversion efficiency

**DOI:** 10.1038/s41598-017-14686-1

**Published:** 2017-10-31

**Authors:** Cheolho Kim, Kiwon Kim, Jun Hyuk Moon

**Affiliations:** Department of Chemical and Biomolecular Engineering, Sogang University, 35 Baekbeom-ro, Mapo-gu, Seoul, 04107 Republic of Korea

## Abstract

Porous carbon spheres (CSs) have distinct advantages in energy storage and conversion applications. We report the preparation of highly monodisperse N-doped microporous CSs through the carbonization of polystyrene-based polymer spheres and subsequent activation. The N-doped microporous CSs have a remarkably high N-doping content, over 10%, and high BET surface area of 884.9 m^2^ g^−1^. We characterize the synergistic effects of the micropores and N doping on the energy storage performance of a supercapacitor electrode consisting of the CSs and on the performance in an electrocatalytic reaction of a CS counter electrode in a photovoltaic cell. The N-doped microporous CSs exhibit a maximum capacitance of 373 F g^−1^ at a current density of 0.2 Ag^−1^, a high capacitance retention up to 62% with a 10-fold increase in current density, and excellent stability over 10,000 charge/discharge cycles. A counter electrode consisting of N-doped microporous CSs was found to exhibit superior electrocatalytic behavior to an electrode consisting of conventional Pt nanoparticles. These CSs derived from polymer spheres synthesized by addition polymerization will be new platform materials with high electrochemical performance.

## Introduction

Carbon nanomaterials is used in electrodes for energy storage and conversion applications such as supercapacitors, lithium-ion batteries, catalytic supports in fuel cells, and photocatalytic and electrocatalytic conversion^1–3^. Of the various nano-carbon morphologies including carbon nanotubes^4^, carbon fibers^5^, and graphene^6^, carbon spheres (CSs) have several distinct advantages. CSs have high packing and tap densities since spheres can be closely packed, and thus can provide high volumetric energy densities^7^. Films consisting of CSs can support well-defined pore networks between the spheres, which facilitates the transport or diffusion of electrolyte ions and thereby enables high kinetic performances^8^. CSs can be prepared in highly concentrated dispersions, whereas the concentrations of other morphologies such as nanotubes or fibers are limited by their large radius of gyration and high aspect ratio. A viscous dispersion with a high concentration is crucial to the preparation of coatings with sufficient thickness for practical applications^9^. Porous CSs are in general prepared via the carbonization of polymer spheres. Phenolic-resin-derived polymer spheres obtained from various precursors including resorcinol, phenol, aminophenol, and other phenol derivatives have been carbonized to obtain CSs^10–13^. Polypyrrole, polysaccharide carrageenan, and polybenzoxazinespheres have also been utilized as CS precursors^14,15^. Such polymer-derived CSs are typically non-porous and dense. Thus, a pore generation process is required; a porous morphology is obtained typically with an activation process that introduces micropores by etching or with a templating process that selectively removes surfactants or block copolymers incorporated in the polymer spheres^16,17^.

Meanwhile, recently, doping with non-metal heteroatoms such as nitrogen (N), fluorine, boron, sulfur, and phosphorus has been demonstrated to be a facile strategy for improving the electrical and electrochemical properties of carbon materials^18–22^. Non-metal atom doping is usually achieved via high-temperature diffusion; in the case of N-doping, the heat-treatment of the carbon sample in the presence of N-rich molecules such as ammonia, carbamide, or melamine results in the diffusion of N atoms into the carbon matrix^22–24^. Non-metal doping of various carbon materials, including graphene and activated carbon particles, as well as carbon nanotubes and fibers, has been performed^5,25–27^. Of the various non-metal heteroatom doping, N doping is highly effective in that it improves the electrochemical pseudocapacitance in various ways. N doping facilitates redox reactions due to the resulting higher positive charge on the carbon atoms adjacent to N atoms^28^. N functional groups (e.g., pyrrolic or pyridinic N) directly induce redox reactions^29^. In particular, the pseudocapacitance provided by N doping is maintained even at high current densities, in contrast to the pseudocapacitance provided by metal oxides or conducting polymers^25,28,30^. Moreover, N doping also enhances electrocatalytic properties: it improves the chemisorption of oxygen, which results in high performances in electrocatalytic oxygen-reduction reactions^14^. Despite these advantages of N doping, there have been few attempts to prepare N-doped CSs. N-enriched porous CSs have been prepared by carbonizing phenolic resin polymer spheres, and were then used in a supercapacitor^31,32^. N-doped microporous CSs have been prepared via the carbonization of polypyrrole and subsequent activation^33^. It is noted that previous N-doped CSs have reported very low N-doping, in particular, low compared to N-doping in carbon nanotubes^11^, carbon nanowires^34^, and graphene^35^, and few studies have emphasized high doping content of around 7 at%.(see Table S1) Moreover, most of the studies have been performed primarily on the electrochemical energy storage properties of N-doped CSs. Thus, a method for the synthesis of CSs with high doping concentrations is still required and applying N-doped CSs to various electrochemical applications remains a challenge.

In this study, we prepared CSs of high N-doping concentration of uniform size of several hundred nanometers, derived from emulsion-polymerized polystyrene (PS)-based spheres. Compared with previous polymer particle precursors, PS particles by emulsion polymerization are easy to synthesize into spherical particles of monodisperse, spherical, and submicrometer. We conducted N-doping along with carbonization of the PS spheres; this approach not only simplifies the process but also enables high N-doping. We achieved a uniform N-doping of the carbon matrix of approximately 10.6 at%. We elucidated the synergistic effects of the micropores and N-doping on the energy storage performance of the CSs in a supercapacitor electrode. The N-doped microporous carbon CSs were found to exhibit a maximum capacitance of 373 F g^−1^ at a current density of 1 A g^−1^. Moreover, we extended the application of the N-doped microporous CSs by using them in the electrocatalytic electrode of a photovoltaic device, i.e., as the counter electrode for a dye-sensitized solar cell (DSSC). The CSs were found to exhibit superior electrocatalytic behavior to conventional Pt nanoparticles. Thus the CSs derived from PS-based materials provide a new platform for high performance electrochemical nanomaterials.

## Results

### Synthesis of Nitrogen-Doped Microporous Carbon Nanospheres and their Characterization

**Figure 1:**
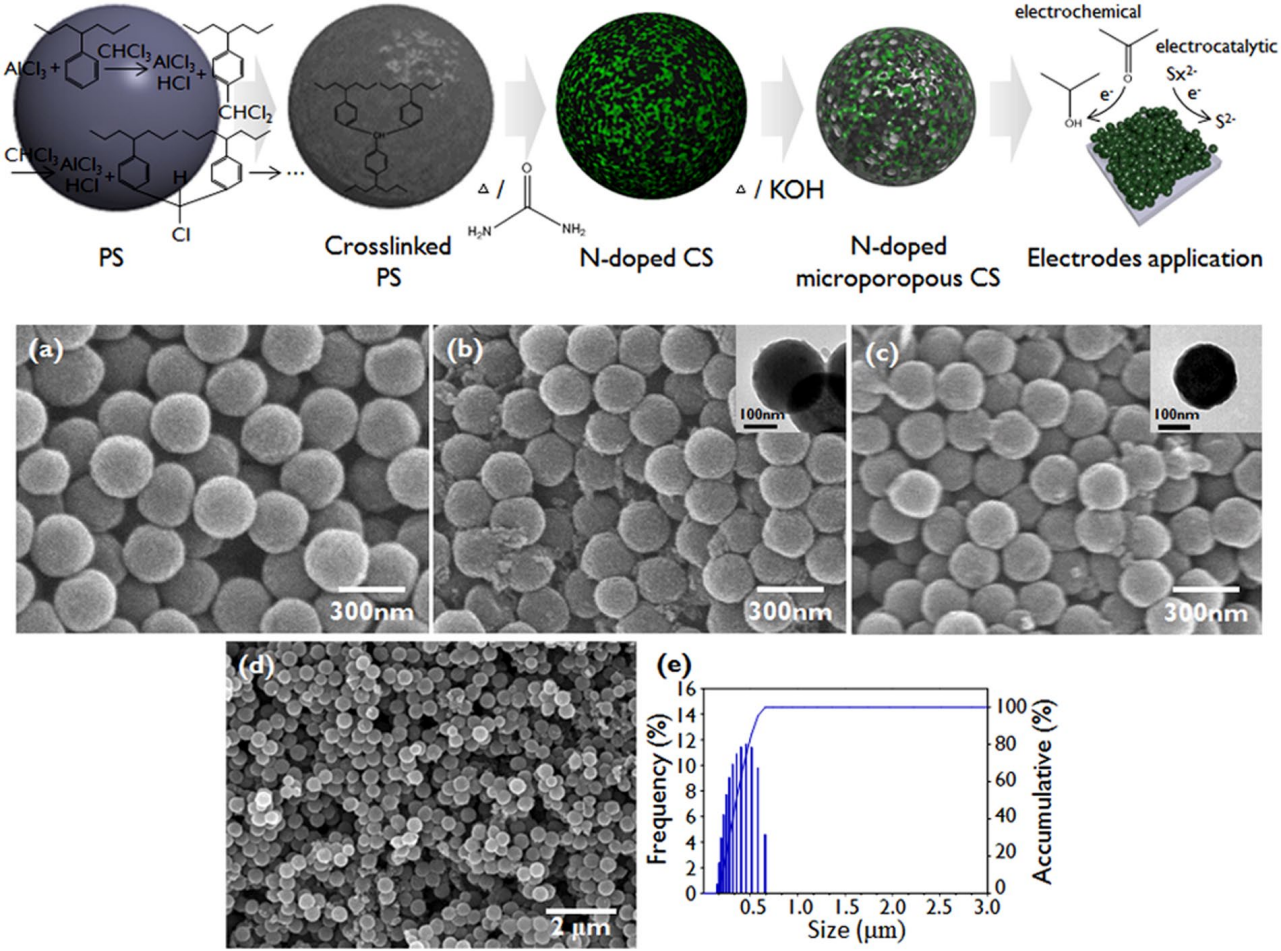
Schematic diagram of the synthesis of N-doped microporous CSs derived from polystyrene-based materials and their applications in electrochemical or electrocatalytic electrodes. SEM images of (**a**) PS spheres, (**b**) N-doped CSs, and (**c**) N-doped microporous CSs. The insets show the TEM images of the samples. (**d**) Low magnification SEM image of N-doped microporous CSs. (**e**) Size-distribution data for the N-doped microporous CSs obtained with light scattering.

The synthesis of N-doped microporous CSs from emulsion-polymerized PS spheres is shown schematically in Fig. 1. The PS spheres were synthesized via the emulsifier-free emulsion polymerization of styrene monomers and subsequently crosslinked via Friedel-Craft alkylation. Crosslinking was required to achieve a high carbon conversion. During the pyrolytic carbonization of the PS spheres, we heat-treated them in the presence of carbamide, resulting in N-doping along with carbonization. It has been reported that carbamide is thermally decomposed and deposited in the form of carbon nitrides, and then the N atoms of the carbon nitrides thermally diffuse inside the carbon lattice at high temperatures (>500 °C)^36^. Note that performing N-doping during pyrolytic carbonization favors a high doping content, as will be discussed later. We then carried out the KOH-assisted activation reaction to introduce micropores into the CSs. The high-temperature KOH treatment of the CSs induces the reaction, 6KOH + 2 C → 2 K + 3H_2_ + 2K_2_CO_3_, and at higher temperatures such as 700 °C the reaction, K_2_CO_3_ + C → K_2_O + 2CO occurs in which C is consumed to create micropores inside the carbon matrix^37^. The XRD results obtained at various temperatures up to 700 °C indicate that K_2_CO_3_ is produced at 600 °C, and at 700 °C K_2_O is produced while K_2_CO_3_ is reduced (see Figure S1).

SEM images of PS spheres, N-doped CSs, and N-doped microporous CSs are shown in Fig. 1. The PS spheres are monodisperse (a polydispersity within 5%) and the diameters of the PS spheres are approximately 285 nm, as shown in Fig. 1a. The diameters of the N-doped CSs are approximately 250 nm, as shown in Fig. 1b, which corresponds to a shrinkage in size of approximately 10%. This size shrinkage is due to the pyrolytic carbonization of PS. The KOH activation maintains the monodispersity of the N-doped CSs but the diameter is decreased further to 230 nm, as observed in Fig. 1c. This decrease in size occurs due to the activation creating pores while etching the carbon matrix. The SEM (Fig. 1d) and DLS data (Fig. 1e) further confirm that the N-doped microporous CSs have a narrow size distribution with particle sizes around 300 nm.

**Figure 2:**
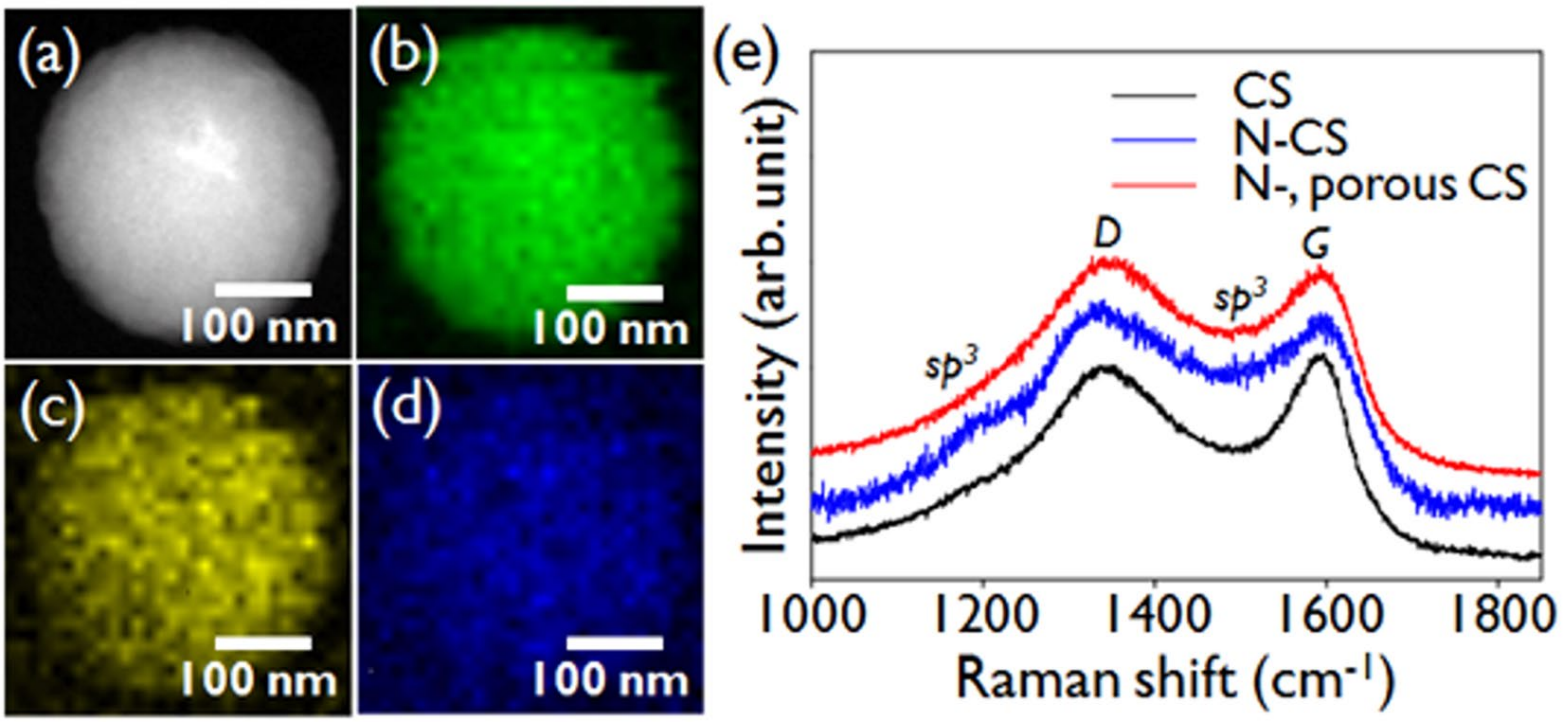
TEM image of (**a**) a N-doped microporous CS, and elemental mapping images of (**b**) carbon, (**c**) nitrogen, and (**d**) oxygen. (**e**) Raman spectra of CSs, N-doped CSs, and N-doped microporous CSs.

The elemental mapping of the N-doped microporous CSs was performed with TEM; a dark-field image and the C, N, and O mappings are shown in Fig. 2b,c and d respectively. The N intensity is higher in the center of the sphere, which confirm that the N doping is not limited to the surface but is uniform over the sphere. The carbon microstructures of the CSs were characterized by recording their Raman spectra. The spectra of N-doped CSs and N-doped microporous CSs contain peaks centered near 1350 cm^−1^ and 1592 cm^−1^, which are the D and G bands respectively, as shown in Fig. 2e. The D band arises from the vibrations of carbon atoms with dangling bonds in plane terminations, and is thus related to the defects of graphitic carbon, whereas the G band arises from the vibrations of sp^2^ carbon atoms in the graphitic layer^38^. In contrast to the results for bare CSs, the N-doped CS and N-doped microporous CS spectra contain additional shoulders near 1180 cm^−1^ and 1500 cm^−1^, which are assigned to sp^3^-hybridized carbon^39^. The presence of these peaks implies that N doping and activation result in the creation of a defective graphitic layer. To quantitatively evaluate the defects, the ratios of the peak intensities of the D and G bands (*I*_*D*_ */I*_*G*_) were compared. The *I*_*D*_ */I*_*G*_ ratios of CSs, N-doped CSs, and N-doped microporous CSs are 0.9733, 1.0172, and 1.0413, respectively. An increase in the *I*_*D*_ */I*_*G*_ ratio is typically due to an increase in the number of sp^2^ crystallite boundaries (i.e., a reduction in the sp^2^ cluster size) or/and an increase in sp^3^ hybridization, which indicates an increase in the proportion of defects^40,41^. We have observed that nitrogen doping increases the micropore, which is considered to be due to the defective site.(see Figure S2) Thus, N doping and pore generation are accompanied by lattice destruction and/or fragmentation. The graphitic crystallite domain sizes were determined by using the Tuinstra-Koenig relationship^42^; the domain sizes of N-doped CSs and N-doped microporous CSs were 16.47 nm and 16.09 nm respectively, while the size of CSs were 16.60 nm, which corresponds well with the above analysis.

**Figure 3:**
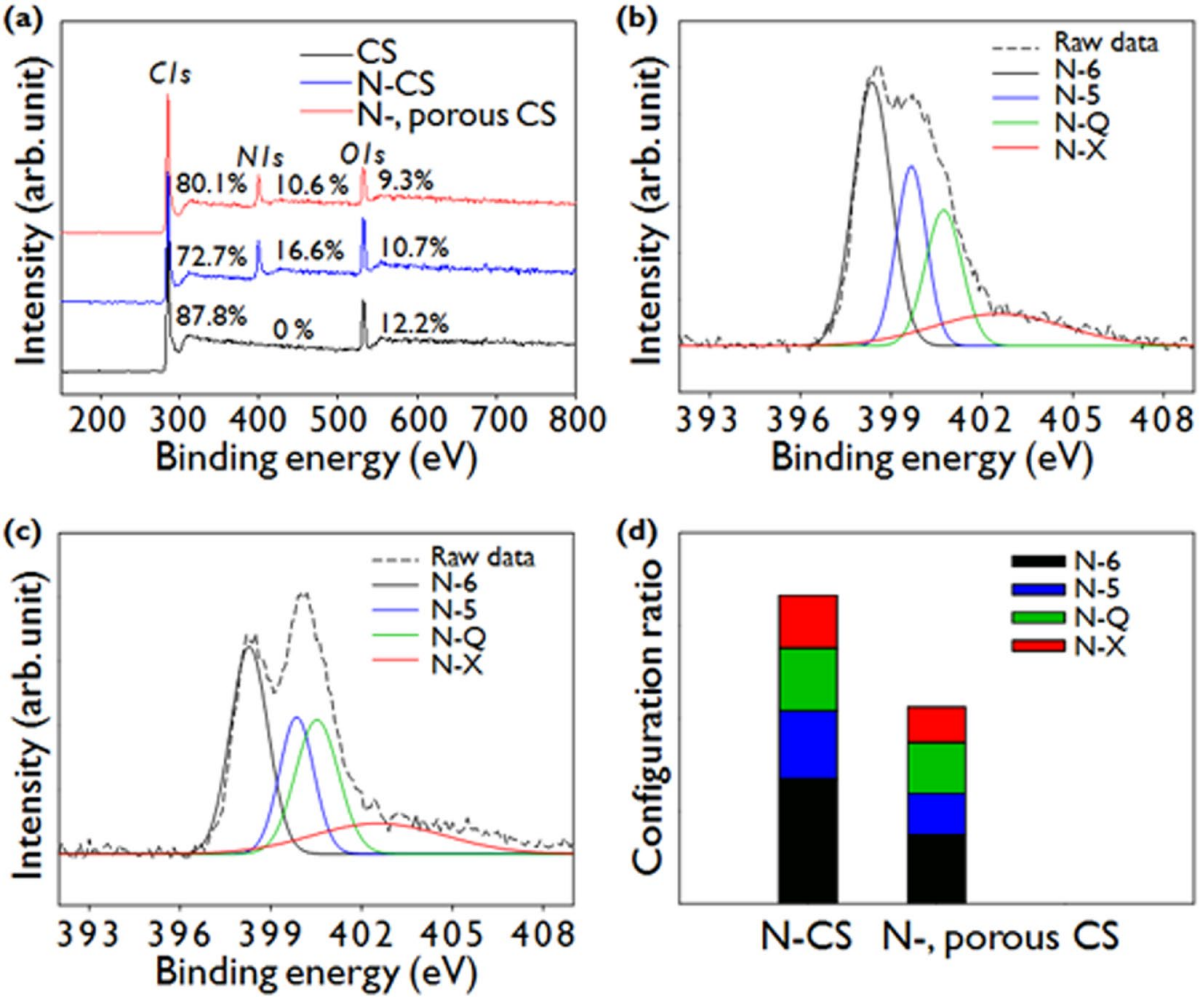
XPS survey spectra of (**a**) CSs, N-doped CSs, and N-doped microporous CSs. High resolution N1s XPS spectra of (**b**) N-CSs, and (**c**) N-doped microporous CSs. (**d**) The corresponding nitrogen configuration ratios.

The chemical compositions of bare CSs, N-doped CSs, and N-doped microporous CSs were characterized with XPS analysis. The XPS survey spectra and the atomic compositions are shown in Fig. 3a. The N contents of N-doped CSs and N-doped microporous CSs are approximately 16 at% and 10 at% respectively. We optimized the N doping content and showed better electrochemical properties at a nitrogen content of 16 at% as shown in Figure S2. Note that the N-doped microporous CSs exhibit a remarkably high N doping content although the N doping content is reduced by the removal of unstable C-N bonds during high temperature activation^43^. Many previous studies that have conducted N doping of various carbon materials such as graphene and carbon nanotubes have reported 4–6% of N-doping. (see Table S2) In addition, N-doping into CSs derived from phenolic resins reported a doping level of 2–5%, with few results highlighting a high doping level of around 7 at%. (see Table S1) Previously, it has been reported that N doping is mediated by oxygenated groups or mostly occurs at defective sites or edges^44,45^. PS-derived CSs contain a high oxygen content of 12.2 at%, as observed in Fig. 3a. The CSs have a high defect density and a small domain size (i.e., a high density of edges) as shown in Raman analysis. The fact that a large amount of oxygenated group and defect generation are accompanied along with the carbonization implies the possibility of high concentration of N-doping.

The N-doping configurations of N-doped CSs and N-doped microporous CSs were further characterized by examining their high resolution N1s spectra, which can be deconvoluted into four peaks located in the regions 398.2–398.6 eV, 399.5–399.7 eV, 400.7–400.8 eV, and 402.5–402.6 eV, as shown in Fig. 3b and c. The first two peaks are attributed to pyridinic-N (sp^2^ hybridized with two carbon atoms, N-6), and pyrrolic N (incorporated in a five-membered ring of carbon atoms, N-5)^46,47^. The peak in the range of 400.7–400.8 eV is due to quaternary nitrogen (sp^3^ hybridized with three carbon atoms, N-Q), i.e. N atoms substituted for carbon atoms in a graphene layer. The peak at around 402.5–402.6 eV is attributed to oxidized N (N-X)^48,49^. The relative proportions of these N configurations in the N-doped CSs and the N-doped microporous CSs are shown in Fig. 3d. The proportions of N-6 and N-5 in the N-doped CSs are larger than those of N-Q and N-X. The proportions of N-6 and N-5 are much lower in the N-doped microporous CSs than in the N-doped CSs. The large decrease in the levels of N-6 and N-5 during the activation step is probably due to their low binding energy, which means that they are likely to be etched during the high-temperature activation reaction^50^.

**Figure 4:**
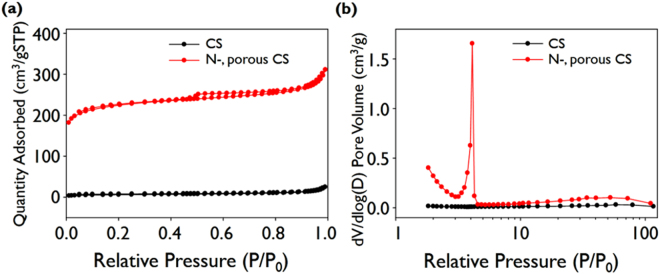
(**a**) BET isotherms and (**b**) pore distributions of CSs and N-doped microporous CSs.

The BET isotherm for N-doped microporous CSs were measured to characterize their pore structures. The BET isotherm for bare CSs was also measured for comparison. The isotherm of N-doped microporous CSs is type I with a steep increase in adsorption at very low relative pressures followed by a plateau and no apparent hysteresis in the adsorption/desorption cycle, as shown in Fig. 4a; these results indicate the presence of abundant micropores and some mesopores. The specific surface areas and total micropore and mesopore volumes for all samples are presented in Table 1. The specific surface area and the total pore volume of N-doped microporous CSs are 844.9 m^2^ g^−1^ and 0.3383 cm^3^ g^−1^ respectively. The BET surface area of the N-doped microporous CSs is approximately 40 times higher than that of bare CSs because of the presence of micropores. The pore size distributions obtained with the Barrett-Joyner-Halenda (BJH) method are also plotted in Fig. 4b: the N-doped microporous CSs contain highly monodisperse micropores with diameters of approximately 4 nm.

**Table 1:** Pore characteristics of CSs and N-doped microporous CSs.

	S_BET_ [m^2^ g^−1^]	S_meso_ [m^2^ g^−1^]	V_total_ [cm^3^ g^−1^]	V_micro_ [cm^3^ g^−1^]	S_meso_ [cm^3^ g^−1^]
CS	20.53	10.32	0.0385	0.00508	0.03341
N-, porous CS	844.9	53.60	0.4815	0.3383	0.1431

### Electrochemical properties and supercapacitor applications

**Figure 5:**
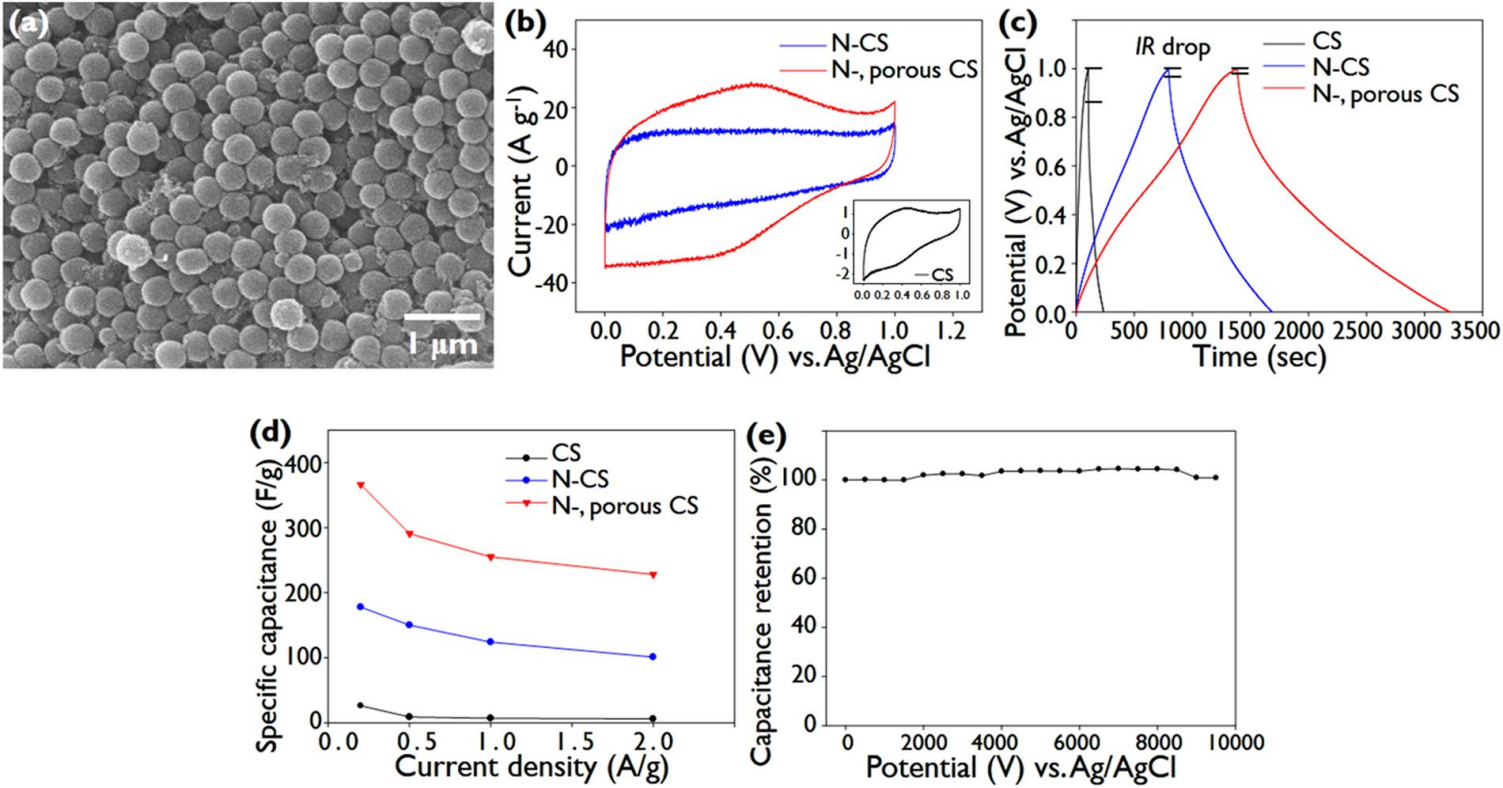
(**a**) SEM image of N-doped microporous CSs. (**b**) Cyclic voltammograms for N-doped CSs and N-doped microporous CSs. The inset shows the cyclic voltammogram for CSs at a scan rate of 10 mV s^−1^. (**c**) Galvanostatic charge-discharge curves for CSs, N-CSs, and N-doped microporous CSs at a current density of 0.2 A g^−1^. (**d**) The specific capacitances of CSs, N-CSs, and N-doped microporous CSs at various current densities. (**e**) Capacitance retention of N-doped microporous CSs measured at a constant current density of 50 A g^−1^. A 1 M H_2_SO_4_ solution was used as the electrolyte solution.

The electrochemical properties of the N-doped microporous CSs were confirmed by cyclic voltammetry (CV) measurement using the film in which the CSs were assembled. (see Fig. 5a) The CV for N-doped microporous CSs as well as those for bare CSs and N-doped CSs are shown in Fig. 5b. The cyclic voltammogram for the bare CSs has a rectangular shape with a hump near 0.4 V, which is typical response of carbonaceous materials containing oxygenated groups; the hump corresponds to the reduction of quinone to hydroquinone in acid solution^51^. Compared to the bare CSs, the N-doped CSs exhibit higher current densities over the entire potential range, which is due to the enhancement of the electric double layer (EDL) capacitance that results from N-doping. Previously, N-doping enhanced the electrical conductivity of carbon, and the EDL capacitance^52^. We observe an increase in conductivity by N doping, as the voltage drop in the charge/discharge measurements. (see Fig. 5c). Meanwhile, compared to CSs, N-doped CSs show higher current densities at electrode potentials less than 0.6 V, as shown in Fig. 5b. The improvement of current densities around 0.2 V has been reported to be a contribution of Faradaic redox capacitance by N-doping; N-doping, particularly in N-5 and N-6 configurations, induces pseudocapacitance via the proton exchange reaction^53^. As demonstrated in Fig. 5d, N-doped CSs contain relatively high proportions of N-6 and N-5 configurations. Further, when compared to the cyclic voltammogram of N-doped CSs, that of the N-doped microporous CSs has a much larger rectangular-like shape and also a more pronounced hump around 0.5 V. This result implies that the activation reaction enhances both the EDL capacitance and the pseudocapacitance.The activation step obviously improves the specific area of the CSs by creating micropores, thereby increasing the EDL capacitance. In addition, the activation step generates more C = O quinone groups as observed in Figure S3, thereby improving the current at around 0.5 V corresponding to the reduction reaction of the quinone group.

The galvanostatic charge/discharge curves of bare CSs, N-doped CSs, and N-doped microporous N-CSs are compared in Fig. 5c. The specific capacitance of each sample was obtained from its charge/discharge curve according to the equation, *C*_*S*_ = (*I* × *∆t*)/(*∆V* × *m*), where *C*_*S*_ is the specific capacitance, *I* is the discharge current, *∆t* is the discharge time, *∆V* is the voltage range, and *m* is the mass of the electrode material^54^. The calculated specific capacitance of the N-doped microporous CSs is 363 F g^−1^ at 0.2 A g^−1^. Note that this specific capacitance of N-doped microporous CSs is the highest level among previous N-doped carbon materials (see Tables S1 and S2). The specific capacitances of the bare CSs and N-doped CSs were calculated to be 30 F g^−1^ and 180 F g^−1^ at 0.2 A g^−1^ respectively. Thus, N-doping enhances the capacitance by a factor of approximately 6 and the subsequent micropore generation further enhances the capacitance by a factor of 2. Meanwhile, the voltage drop (or internal resistance drop) is decreased by N-doping and subsequent micropore generation; the voltage drops for bare CSs, N-doped CSs, and N-doped microporous CSs are 110 mV, 22 mV, and 12 mV, respectively, as shown in Fig. 5c. This decrease probably arises because activation increases the specific area of the CSs without any substantial decrease in the N-doping content.

The specific capacitances were calculated at various current densities in the range 0.2 to 2 A g^−1^: there is a decrease in the capacitance as the current density increases, as shown in Fig. 5d. The N-doped microporous CSs exhibit a specific capacitance of 373 F g^−1^ at 0.2 A g^−1^ and a capacitance retention of 61% when current density was increased 10-fold, whereas the capacitance retentions of the CSs and N-doped CSs are 23% and 56% respectively. Note that the capacitance retention of the N-doped microporous CSs is comparable to that of the N-doped CSs as well as that of bare CSs. It has often been observed that the presence of micropores, particularly those smaller than a few nm in size, impairs the retention at high current densities due to their limited ion transport kinetics^55,56^. In our case, the activation reaction creates large micropores with sizes near 4 nm, and moreover the well-defined pore network within the highly monodisperse, sub-micrometer-size CS assembly could facilitate ion transport. Further, considering that capacitance retention is markedly lower in high resistance electrodes, the high retention of N-doped microporous CSs is attributed to their higher conductivity because their N-doping content is high even after the activation reaction, as is evident in the voltage drop analysis. The cycle stability of the N-doped microporous CSs was also assessed, as shown in Fig. 5e. The capacitance retention is excellent even after 10,000 cycles, i.e. the capacitance is still 98% of the initial capacitance, which confirms the high performance of the N-doped microporous CSs as a supercapacitor electrode.

### Electrocatalytic applications

**Figure 6:**
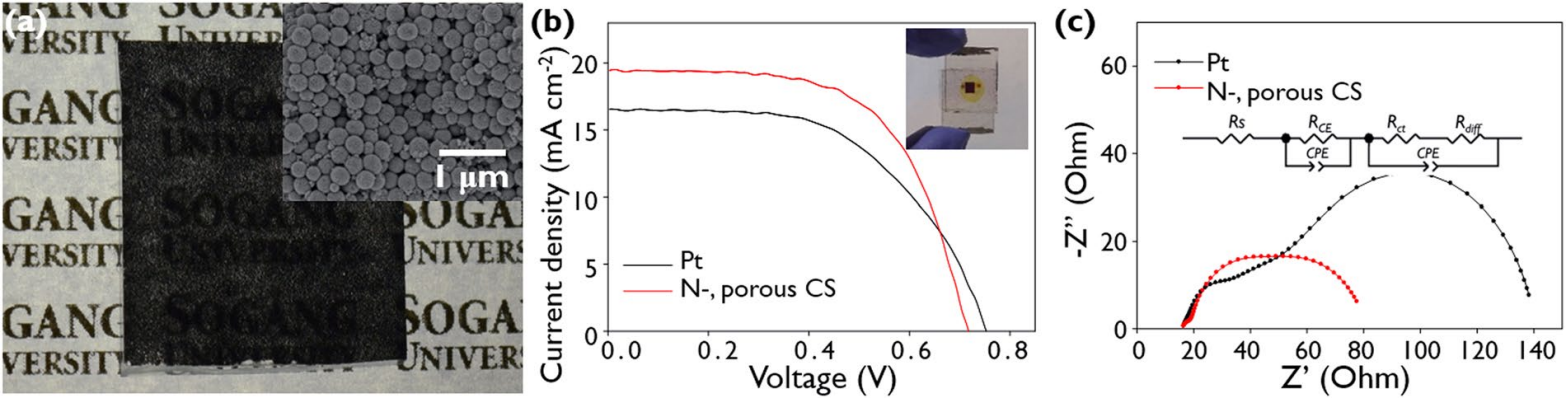
(**a**) Digital image of the N-doped microporous CS CE. The inset shows a SEM image of the N-doped microporous CS CE. (**b**) Photocurrent-voltage curves for the DSSCs with conventional Pt and N-doped microporous CS CEs. The inset shows a digital image of the DSSC containing the N-doped microporous CS CE. (**c**) Nyquist plots for the DSSCs. The inset shows the corresponding equivalent circuit model.

We tested the N-doped microporous CSs in an electrocatalysis application, i.e. as a counter electrode (CE) in a dye-sensitized solar cell (DSSC). The conventional DSSC was composed of a dye-sensitized TiO_2_ photoanode, a platinum (Pt) CE, and an electrolyte solution containing a redox ion couple (I^−^/I^3−^ redox ion pairs). The CE of the DSSC plays the role of the electrocatalytic regeneration of the redox ion couple that is oxidized at the photoanode^57^, i.e., the CE induces the electrocatalytic reduction of the oxidized redox ion, I^3−^ into I^−^. Pt has been widely used in CEs because of its high electrocatalytic activity in regeneration reactions and high conductivity, but the high costs and scarcity of Pt limit its practical applications^58^. As an alternative, carbon materials such as carbon nanotubes, graphene, and activated carbon are attractive because of their high conductivities, large surface areas, and high corrosion resistance towards redox ions as well as their low cost^59–62^. Here, the N-doped microporous CS-based CE was prepared by coating the CSs onto a transparent conductive substrate, as shown in Fig. 6a. The CE was assembled with the conventional TiO_2_ photoanode to fabricate the DSSC (see inset picture of Fig. 6b). Here, disulfide (S^2−^/S_x_^2−^) ions were used in the electrolyte instead of the conventional I^−^/I^3−^ ions. The disulfide redox couple is a promising electrolyte for high performance DSSCs because it possesses a higher redox potential than the I^−^/I^3−^ redox couple, is non-corrosive toward dyes, and exhibits negligible visible-light absorption^63,64^.

The photocurrent density vs applied voltage curve for the DSSC containing a N-doped microporous CS CE is shown in Fig. 6b. The results for a DSSC with a conventional Pt-CE are also shown for comparison. In Figure S4, the cyclic voltammogram of the disulfide electrolyte of the two counter electrodes was measured and the result shows a similar redox curve. The photovoltaic parameters of the DSSC, including *J*_*sc*_ (short-circuit current), *V*_*oc*_ (open-circuit voltage), the fill factor (*FF*), and the overall conversion efficiency (*η*) obtained by (*J*_*sc*_ *V*_*oc*_ *FF*/1000 W m^−2^) are also listed in Table 2. The *η* value for the DSSC with a N-doped microporous CS CE was found to be 8.621% whereas that of the DSSC with a conventional Pt CE was 6.884%. Thus, the CS electrode DSSC exhibits a 25% higher *η* than the Pt electrode DSSC, which is attributed to the higher *J*_*sc*_ and *FF* of the N-doped microporous CS DSSC. Many previous studies have reported that the *η* values of various carbon CEs for DSSCs are comparable to those of Pt CEs.(see Table S3). Thus, the present result of the N-doped microporous CS CE demonstrate its superior electrocatalytic properties.

**Table 2:** Photovoltaic parameters for the DSSCs with conventional Pt and N-doped microporous CS CEs.

	*J*_*SC*_ [mA cm^−2^]	*V*_*OC*_ [V]	*FF*	*ƞ* [%]
Pt	16.55	0.751	0.554	6.884
N-doped, porous CS	19.42	0.711	0.624	8.621

The electrochemical impedance spectra (EIS) of the N-doped microporous CS electrode and Pt electrode DSSCs were recorded to characterize their charge transfer processes. Three-overlapped semicircles are evident in the Nyquist plots, as shown in Fig. 6c; the semicircles in the high, middle, and low frequency regions correspond to the charge transfer resistance at the electrolyte/CE interface, i.e., the electrocatalytic reaction resistance (*R*_*CE*_), the resistance at the TiO_2_/dye/electrolyte interface (*R*_*ct*_), and the diffusion impedance of the redox electrolyte (*R*_*diff*_), respectively^65,66^. To analyze the Nyquist plot, we used an equivalent circuit shown in the inset of Fig. 6c. Note in particular that the *R*_*CE*_ of the CS CE is much smaller than that of the Pt CE (see Table S4). This result indicates that the electrochemical reaction kinetics of the disulfide redox ions on the N-doped microporous CS CE are much enhanced when compared to those on the Pt CE. This high electrocatalytic activity of the CS CE may be attributed to highly defective graphitic character and abundant oxygen/nitrogen functional groups of the N-doped microporous CSs^67^. It has been known that the functional group near the carbon crystal edge are known to be the dominant catalytic active sites for oxidized disulfide^68,69^. Moreover, the hierarchical, well-defined pores of the CS-assembled film may facilitate ion transport, which improves the electrocatalytic reaction. The high *R*_*ct*_ decreases the rate of dye regeneration and enhances the charge recombination reaction of the oxidized ions. Thus, the higher *J*_*sc*_ of the N-doped microporous CS DSSC could be explained by its lower *R*_*CE*_^68^. Further, the *R*_*ct*_ of the N-doped microporous CS CE is 15% smaller than that of the Pt CE: we also measured the ohmic series resistance (*R*_*S*_) with a 4-point probe, and found that the *R*_*S*_ of the CS CE is slightly lower that of the Pt CE, as shown in Table S5. This low resistance of the CS film is probably due to the high N-doping. The high *FF* of the N-doped microporous CS DSSC is thus explained by the low internal resistance of the CS film^68^.

## Discussion

The preparation of microporous, heteroatom-doped CSs is a synergistic strategy for improving their energy storage/conversion efficiency. The heteroatom doping ameliorates their performance in electrochemical and electrocatalytic reactions, and the introduction of micropores maximizes their specific areas and thereby the reaction throughput. Although porous CSs with heteroatom doping have been prepared from phenolic-resin-derived polymer spheres, they have typically been prepared for energy storage applications (i.e., supercapacitors), and CSs with high doping levels were hardly synthesized. We obtained highly monodisperse N-doped microporous CSs by performing the carbonization of PS-based spheres and a subsequent activation reaction. The N-doping content of the N-doped microporous CSs is above 10% because doping was performed simultaneously with carbonization and the polystyrene-derived carbon has a highly defective microstructure. In applications as supercapacitor electrodes, these N-doped microporous CSs were found to provide a maximum capacitance of 373 F g^−1^ at a current density of 0.2 A g^−1^; they also exhibit high capacitance retention and excellent cycle performance. When used in the electrocatalytic electrodes of a DSSC, the N-doped microporous CSs were found to exhibit superior electrocatalytic behavior to a conventional Pt electrode. We believe that various polymer spheres synthesized by addition polymerization will be a platform for synthesizing nanocarbon materials with high performance electrochemical and electrocatalytic nanomaterials.

## Methods

### Synthesis of Polystyrene-Derived Carbon Spheres

Synthesis of Polystyrene-Derived Carbon Spheres. Monodisperse polystyrene-based polymer spheres were synthesized by performing the emulsifier-free emulsion polymerization of styrene monomer (99.9% Sigma-Aldrich) in the presence of methyl methacrylate (99.9% Sigma-Aldrich, MMA) monomer (approximately 5 wt% with respect to styrene). Styrene and MMA were vigorously mixed in water along with 5 wt% potassium persulfate initiator (Aldrich), and then 40 wt% divinyl benzene (Aldrich) was added. After overnight polymerization, the polymer colloids were washed with water several times and re-dispersion in deionized water. Subsequently, the polystyrene (PS) was further crosslinked via Friedel-Crafts alkylation. The PS was dispersed in chloroform solution in which 8 wt% of anhydrous aluminium chloride was contained, and the reaction was carried out at 60 °C for 24 hours. The resulting crosslinked polystyrene PS spheres were purified by washing with ethanol. N-doped CSs were prepared by the high temperature carbonization of the PS in the presence of carbamide; the cross-linked PS spheres were mixed with carbamide powder (Aldrich) at a mixing weight ratio of PSs to carbamide of 1:50, and then heated to 700 °C for 2 h under an argon atmosphere. The activation (i.e., micropore generation) of the N-doped CSs was achieved by treating them with KOH solution. The N-doped CSs were mixed with 45 wt% KOH aqueous solution, with the mass ratio of N-doped CS: KOH controlled at 1:1, and then dried in an oven at 90 °C. The mixture was heated to 700 °C for 2 h. The N-doped microporous CSs were washed with HCl and distilled water several times.

### Characterization

The morphologies were determined with a scanning electron microscope (Hitachi, S-4700) and transmission electron microscope (Carl Zeiss, LIBRA 120, 80 kV). Energy dispersive spectroscopy (EDS) elemental mapping was performed with a transmission electron microscope (JEOL, JEM-2100F, 200 kV). X-Ray photoelectron spectroscopy (XPS, ESCALAB 250 XPS) was performed for elemental analysis by using a Al Ka X-ray source at a pressure of 1 × 10^−10^ torr. The Raman spectra were collected by using a Horiba Jobin Yvon LabRAM HR equipped with an air-cooled Ar ion laser operated at 541 nm.

### Electrochemical characterization

A three electrode system was used to measure the electrochemical properties. To fabricate CS working electrode, CSs were dispersed in a Nafion solution (Sigma-Aldrich) and anhydrous ethanol (Sigma-Aldrich) and this solution was dropped onto a glassy carbon electrode. A platinum wire and an Ag/AgCl (3 M NaCl) electrode were used as the counter and reference electrodes, respectively. A 1 M H_2_SO_4_ solution was prepared as an electrolyte. Cyclic voltammetry (CV) and galvanostatic charge–discharge cycles were recorded with a VersaSTAT (AMETEK). CV was measured with a potential range of 0–1 V versus an Ag/AgCl (3 M NaCl) electrode with a range of scan rates (5–200 mV s^−1^). The galvanostatic charge/discharge measurements were measured with a voltage range of 0–1 V at a constant current of 0.2–1 A g^−1^.

### Electrocatalytic characterization

N-doped microporous CSs were tested by fabricating a counter electrode (CE) for use in a DSSC. The CE was prepared by coating the N-doped microporous CSs onto a FTO substrate. Briefly, CSs were dispersed in a PVdF N-methyl-2-pyrrolidone solution, and the solution was cast onto a FTO substrate and bladed to create a film with uniform thickness. The thickness of the CS electrode film was approximately 10 μm. A conventional Pt CE was prepared by coating a FTO substrate with a 0.5 mM H_2_PtCl_6_ solution in anhydrous ethanol followed by heat treatment at 450 °C for 30 min. To fabricate the photoanode for the DSSC, a nanocrystalline TiO_2_ suspension (Dyesol, TiO_2_ Paste DSL 18NR-T) was screen printed followed by heat treatment at 500 °C for 15 min. The TiO_2_ anode was sensitized by immersion in a dye solution (0.5 M ruthenium-535-bis-TBA ethanol solution, Solaronix, N719) for 18 h at room temperature. Finally, the electrolyte solution was injected into the gap in the CE and photoanode assembly. The electrolyte solution was prepared by dissolving polysulfide with tetramethylammonium sulfide in an 8.5:1.5 (v/v) ratio with a mixture of acetonitrile (Aldrich) and valeronitrile (Aldrich). The polysulfide with tetra-methylammonium sulfide was synthesized as reported elsewhere^70^.

## Electronic supplementary material

Supplementary information
