# Supplementary material for: Highly N-doped microporous carbon nanospheres with high energy storage and conversion efficiency
Source: Sci Rep. 2017 Oct 31;7:14400. doi: 10.1038/s41598-017-14686-1 (PMC5663965; doi:10.1038/s41598-017-14686-1)
Supplement: Supplementary file 1 — Supplementary information [file 41598_2017_14686_MOESM1_ESM.doc]

Supplementary information

Highly N-doped microporous carbon nanospheres with high energy storage and conversion efficiency

Cheolho Kim, Kiwon Kim, and Jun Hyuk Moon,*

Department of Chemical and Biomolecular Engineering, Sogang University
1 Sinsu-dong, Mapo-gu, Seoul, 04107, Republic of Korea

Corresponding author, E-mail: [junhyuk@sogang.ac.kr](mailto:junhyuk@sogang.ac.kr)


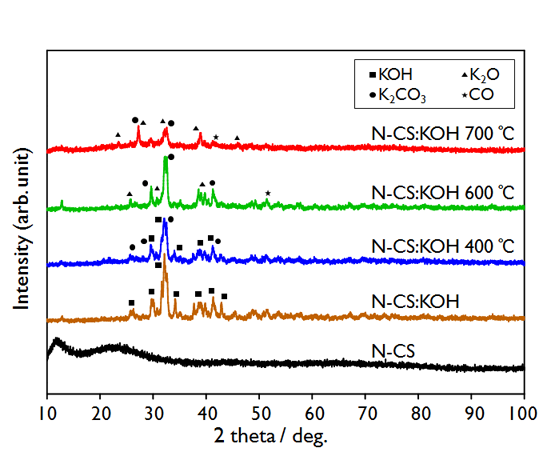


**Figure S1.** XRD patterns of N-CS and N-doped microporous CS during the reaction with KOH at different temperatures


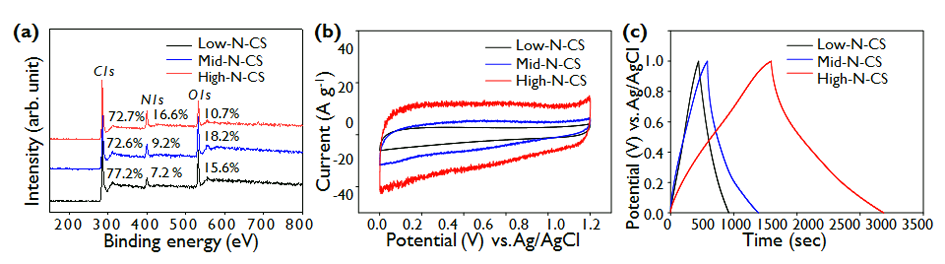


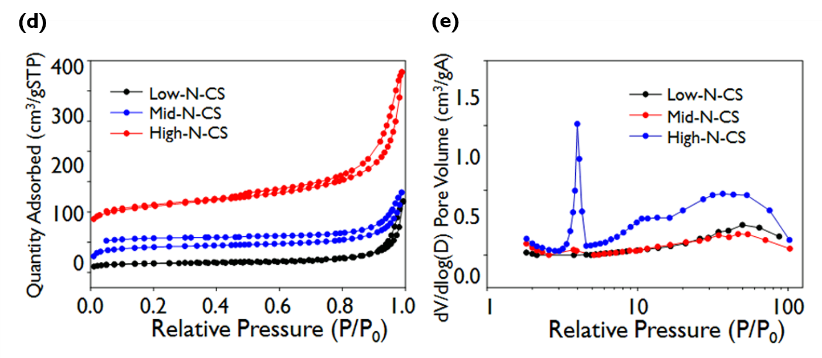


**Figure S2**. (a) XPS spectra of Low-, Mid-, High-N-CSs at a scan rate of 10 mV s^-1^. (b) Cyclic voltammograms and (c) galvanostatic charge-discharge curves for Low-, Mid-, High-N-CSs. (d) BET isotherms and (e) pore-size distributions of Low-, Mid- and High-N-CSs.

We controlled the urea precursor when carbonizing, producing N-CS containing 7.2, 9.2, and 16.6% N content, respectively. Each sample was named Low-, Mid-, and High-N-CS, respectively. (a) XPS spectra of Low-, Mid-, High-N-CSs at a scan rate of 10 mV s^-1^. (b) Cyclic voltammograms and (c) galvanostatic charge-discharge curves for Low-, Mid-, High-N-CSs.

We studied whether nitrogen doping affects porosity. We synthesized N-CS containing 7.2%, 9.2%, and 16.6% nitrogen. Each named Low-, Mid-, and High-N-CS. As the nitrogen doping increased, we found that the BET surface area increased. Specifically, Low-, Mid-, and High-N-CS showed specific surface areas of 21, 63 and 162 m^2^ / g, respectively. This is due to the large increase in micropores as seen from the pore size distribution. When nitrogen doping is performed, many defects are generated in the carbon lattice, which can be understood as introducing a micropore.


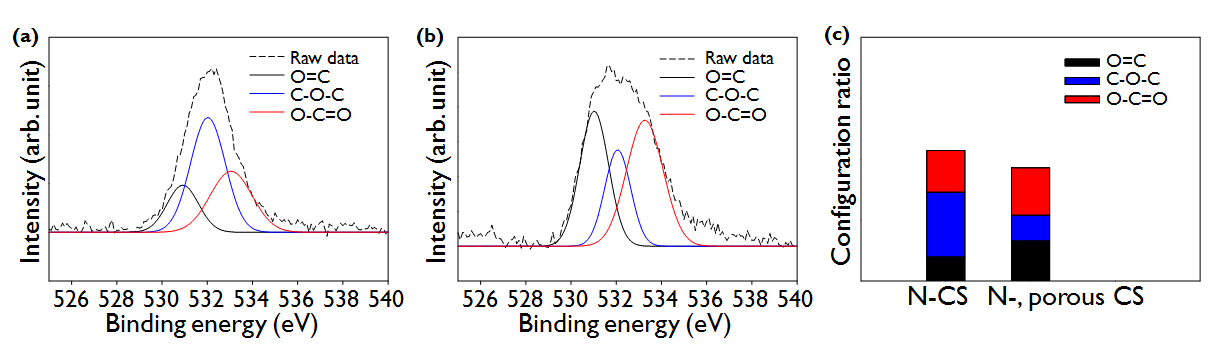


**Figure S3.** High resolution O1s XPS spectra of (a) N-CSs and (b) N-doped microporous CSs. (d) The corresponding oxygen configuration ratios


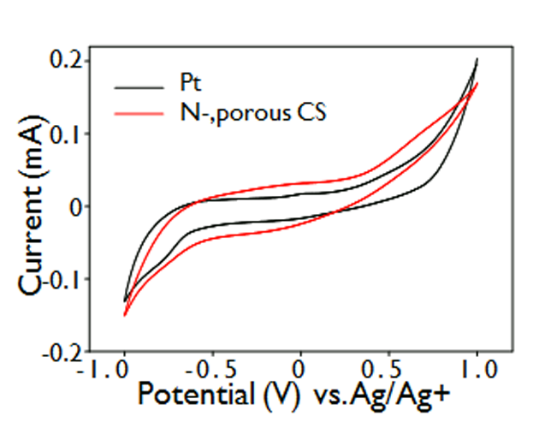


**Figure S4**. Cyclic voltammogram (CV) curves of Pt and N-, porous CS electrodes at a scan rate of 20 mV/s in sulfide-based electrolyte.

**Table S1.** Summary of the comparative electrochemical performance and N contents of N-doped carbon spherical particles.

| Materials | Specific capacitance | Nitrogen content | Reference |
| --- | --- | --- | --- |
| N-doped, porous CS | 373 F g^-1^ at 0.2 A g^-1^  290 F g^-1^ at 0.5 A g^-1^  255 F g^-1^ at 1.0 A g^-1^ | 10.6 % | Our works |
| Nitrogen-doped hierarchical porous CS | 233 F g^-1^ at 0.2 A g^-1^ | 5.0 % | [^1^](#_ENREF_1) |
| Nitrogen doped mesoporous CS | 159 F g^-1^ at 1.0 A g^-1^ | 2.16 % | [^2^](#_ENREF_2) |
| Nitrogen-doped hollow CS | 122 F g^-1^ at 0.5 A g^-1^ | 7.2 % | [^3^](#_ENREF_3) |
| Nitrogen-doped porous CS | 194.7 F g^-1^ at 0.5 Ag^-1^ | 3.25 % | [^4^](#_ENREF_4) |

**Table S2.** Summary of the comparative electrochemical performance and nitrogen contents of N-doped carbon materials.

| Materials | Specific capacitance | Nitrogen content | Reference |
| --- | --- | --- | --- |
| N-doped, porous CS | 373 F g^-1^ at 0.2 A g^-1^  290 F g^-1^ at 0.5 A g^-1^  255 F g^-1^ at 1.0 A g^-1^ | 10.6 % | Our works |
| Nitrogen-doped hierarchical porous carbon | 260.5 F g^-1^ at 0.2 A g^-1^ | 0.7 % | [^5^](#_ENREF_5) |
| Nitrogen doped graphene | 289 F g^-1^ at 0.2 A g^-1^ | 5.63 % | [^6^](#_ENREF_6) |
| Nitrogen-doped electrospun carbon nanofiber | 231 F g^-1^ at 0.2 A g^-1^ | 5.26 % | [^7^](#_ENREF_7) |
| Porous nitrogen-doped graphene/CNTs composite | 246.6 F g^-1^ at 0.5 A g^-1^ | 4.12 % | [^8^](#_ENREF_8) |
| Nitrogen doped carbon nanowires | 207 F g^-1^ at 1.0 A g^-1^ | 5.59 % | [^9^](#_ENREF_9) |
| N-doped nanoporous carbon | 250 F g^-1^ at 0.5 A g^-1^ | 5.37 % | [^10^](#_ENREF_10) |
| Nitrogen-doped porous carbon derived from biomass waste | 255 F g^-1^ at 0.5 A g^-1^ | 6.2 % | [^11^](#_ENREF_11) |

**Table S3.** Summary of the comparative electrochemical performance and nitrogen contents of N-doped carbon materials.

| Materials | ƞ  [%] | ƞ (Pt)  [%] | Reference |
| --- | --- | --- | --- |
| Activated N-doped porous carbon | 6.9 | 7.1 | [^12^](#_ENREF_12) |
| Activated carbon | 3.89 | 4.30 | [^13^](#_ENREF_13) |
| Conductive carbon | 6.7 | 7.5 | [^14^](#_ENREF_14) |
| Carbon dye | 7.5 | 7.5 | [^14^](#_ENREF_14) |
| Carbon fiber | 2.7 | 4.75 | [^15^](#_ENREF_15) |
| CNTs | 7.67 | 7.83 | [^16^](#_ENREF_16) |
| Mesoporous carbon | 6.18 | 6.26 | [^17^](#_ENREF_17) |
| Ordered mesoporous carbon | 7.5 | 7.5 | [^14^](#_ENREF_14) |
| Pen ink | 6.18 | 6.75 | [^18^](#_ENREF_18) |
| Hollow Cf | 7.21 | 7.69 | [^19^](#_ENREF_19) |
| VASWCNTs | 5.25 | 3.49 | [^20^](#_ENREF_20) |
| Bucky paper | 4.02 | 4.08 | [^21^](#_ENREF_21) |
| Graphene | 4.99 | 5.48 | [^22^](#_ENREF_22) |
| Graphene | 5.73 | 6.89 | [^23^](#_ENREF_23) |
| Graphene | 9.3 | 8.1 | [^24^](#_ENREF_24) |
| Graphene | 9.54 | 9.14 | [^25^](#_ENREF_25) |
| Carbon paste | 6.71 | 7.06 | [^26^](#_ENREF_26) |
| Ordered mesoporous carbon | 8.11 | 8.16 | [^27^](#_ENREF_27) |
| Transparent carbon | 6.07 | 6.89 | [^28^](#_ENREF_28) |

**Table S4.** Electrochemical parameters of DSSCs with conventional Pt-CE and DSSCs containing N-doped microporous CS CE.

| Materials | *R_CE_* | *R_ct_* |
| --- | --- | --- |
| Pt | 27.56 | 26.24 |
| N-doped,porous CS | 4.119 | 23.28 |

**Table S5.** Surface resistance of Pt-CE and N-doped microporous CS CE.

| Materials | Resistance  [kΩ/□] |
| --- | --- |
| Pt | 6.629 |
| N-doped,porous CS | 6.581 |

**References**

1. Jiang, J. *et al.* Nitrogen-doped hierarchical porous carbon microsphere through KOH activation for supercapacitors. *J. Colloid Interf. Sci.* **452**, 54-61 (2015).

2. Chen, A. *et al.* Controllable synthesis of nitrogen-doped hollow mesoporous carbon spheres using ionic liquids as template for supercapacitors. *Appl. Surf. Sci.* **393**, 151-158 (2017).

3. Liu, L. *et al.* Nitrogen-doped hollow carbon spheres with a wrinkled surface: their one-pot carbonization synthesis and supercapacitor properties. *Chem. Commun.* **52**, 11693-11696 (2016).

4. Chen, X. Y., Chen, C., Zhang, Z. J. & Xie, D. H. Nitrogen-Doped Porous Carbon Spheres Derived from Polyacrylamide. *Ind. Eng. Chem. Res.* **52**, 12025-12031 (2013).

5. Zhou, J. *et al.* Nitrogen-doped hierarchical porous carbon materials prepared from meta-aminophenol formaldehyde resin for supercapacitor with high rate performance. *Electrochim. Acta* **153**, 68-75 (2015).

6. Haque, E. *et al.* Nitrogen doped graphene via thermal treatment of composite solid precursors as a high performance supercapacitor. *RSC Adv.* **5**, 30679-30686 (2015).

7. Tian, X. *et al.* Synthesis of nitrogen-doped electrospun carbon nanofibers with superior performance as efficient supercapacitor electrodes in alkaline solution. *Electrochim. Acta* **185**, 40-51 (2015).

8. Lin, T.-T., Lai, W.-H., Lü, Q.-F. & Yu, Y. Porous nitrogen-doped graphene/carbon nanotubes composite with an enhanced supercapacitor performance. *Electrochim. Acta* **178**, 517-524 (2015).

9. Zhao, S. *et al.* High capacity and high rate capability of nitrogen-doped porous hollow carbon spheres for capacitive deionization. *Appl. Surf. Sci.* **369**, 460-469 (2016).

10. Tang, H. *et al.* Octa(aminophenyl)silsesquioxane derived nitrogen-doped well-defined nanoporous carbon materials: Synthesis and application for supercapacitors. *Electrochim. Acta* **194**, 143-150 (2016).

11. Ma, G. *et al.* Nitrogen-doped porous carbon derived from biomass waste for high-performance supercapacitor. *Bioresource Technol.* **197**, 137-142 (2015).

12. Wang, L. *et al.* Nitrogen-Doped Porous Carbons As Electrode Materials for High-Performance Supercapacitor and Dye-Sensitized Solar Cell. *ACS Appl. Mater. Inter.* **7**, 20234-20244 (2015).

13. Imoto, K. *et al.* High-performance carbon counter electrode for dye-sensitized solar cells. *Sol. Energy Mater. Sol. Cells* **79**, 459-469 (2003).

14. Wu, M., Lin, X., Wang, T., Qiu, J. & Ma, T. Low-cost dye-sensitized solar cell based on nine kinds of carbon counter electrodes. *Energy Environ. Sci.* **4**, 2308-2315 (2011).

15. Hou, S. *et al.* Transparent conductive oxide-less, flexible, and highly efficient dye-sensitized solar cells with commercialized carbon fiber as the counter electrode. *J. Mater. Chem.* **21**, 13776-13779 (2011).

16. Lee, W. J., Ramasamy, E., Lee, D. Y. & Song, J. S. Efficient Dye-Sensitized Solar Cells with Catalytic Multiwall Carbon Nanotube Counter Electrodes. *ACS Appl. Mater. Inter.* **1**, 1145-1149 (2009).

17. Wang, G., Xing, W. & Zhuo, S. Application of mesoporous carbon to counter electrode for dye-sensitized solar cells. *J. Power Sources* **194**, 568-573 (2009).

18. Cai, X., Lv, Z., Wu, H., Hou, S. & Zou, D. Direct application of commercial fountain pen ink to efficient dye-sensitized solar cells. *J. Mater. Chem.* **22**, 9639-9644 (2012).

19. Park, S.-H., Kim, B.-K. & Lee, W.-J. Electrospun activated carbon nanofibers with hollow core/highly mesoporous shell structure as counter electrodes for dye-sensitized solar cells. *J. Power Sources* **239**, 122-127 (2013).

20. Hao, F. *et al.* High Electrocatalytic Activity of Vertically Aligned Single-Walled Carbon Nanotubes towards Sulfide Redox Shuttles. *Sci. Rep.* **2**, 368 (2012).

21. Roy, S. *et al.* Plasma modified flexible bucky paper as an efficient counter electrode in dye sensitized solar cells. *Energy Environ. Sci.* **5**, 7001-7006 (2012).

22. Roy-Mayhew, J. D., Bozym, D. J., Punckt, C. & Aksay, I. A. Functionalized Graphene as a Catalytic Counter Electrode in Dye-Sensitized Solar Cells. *ACS Nano* **4**, 6203-6211 (2010).

23. Kavan, L., Yum, J. H. & Grätzel, M. Optically Transparent Cathode for Dye-Sensitized Solar Cells Based on Graphene Nanoplatelets. *ACS Nano* **5**, 165-172 (2011).

24. Kavan, L., Yum, J.-H., Nazeeruddin, M. K. & Grätzel, M. Graphene Nanoplatelet Cathode for Co(III)/(II) Mediated Dye-Sensitized Solar Cells. *ACS Nano* **5**, 9171-9178 (2011).

25. Xu, X. *et al.* Electrochemically Reduced Graphene Oxide Multilayer Films as Efficient Counter Electrode for Dye-Sensitized Solar Cells. *Sci. Rep.* **3**, 1489 (2013).

26. Gao, Y. *et al.* Improvement of adhesion of Pt-free counter electrodes for low-cost dye-sensitized solar cells. *J. Photochem. Photobiol. A: Chem.* **245**, 66-71 (2012).

27. Wang, C. *et al.* A low-cost bio-inspired integrated carbon counter electrode for high conversion efficiency dye-sensitized solar cells. *Phys. Chem. Chem. Phys.* **15**, 14182-14187 (2013).

28. Bu, C. *et al.* Highly Transparent Carbon Counter Electrode Prepared via an in Situ Carbonization Method for Bifacial Dye-Sensitized Solar Cells. *ACS Appl. Mater. Inter.* **5**, 7432-7438 (2013).
